# Spin-orbit coupling suppression and singlet-state blocking of spin-triplet Cooper pairs

**DOI:** 10.1126/sciadv.abe0128

**Published:** 2021-01-13

**Authors:** Sachio Komori, James M. Devine-Stoneman, Kohei Ohnishi, Guang Yang, Zhanna Devizorova, Sergey Mironov, Xavier Montiel, Linde A. B. Olde Olthof, Lesley F. Cohen, Hidekazu Kurebayashi, Mark G. Blamire, Alexandre I. Buzdin, Jason W. A. Robinson

**Affiliations:** 1 Department of Materials Science & Metallurgy, University of Cambridge, 27 Charles Babbage Road, Cambridge CB3 0FS, UK.; 2 Department of Physics, Kyushu University, 744 Motooka, Fukuoka 819-0395, Japan.; 3 Research Center for Quantum Nano-Spin Sciences, 744 Motooka, Fukuoka 819-0395, Japan.; 4 Moscow Institute of Physics and Technology, Dolgoprudny 141700, Russia.; 5 Kotelnikov Institute of Radio-engineering and Electronics RAS, Moscow 125009, Russia.; 6 Institute for Physics of Microstructures, Russian Academy of Sciences, GSP-105, Nizhny Novgorod 603950, Russia.; 7 The Blackett Laboratory, Imperial College London SW7 2AZ, UK.; 8 London Centre for Nanotechnology and Department of Electronic and Electrical Engineering at University College London, London WC1H 01H, UK.; 9 University Bordeaux, LOMA UMR-CNRS 5798,, F-33405 Talence Cedex, France.; 10 Sechenov First Moscow State Medical University, Moscow 119991, Russia.

## Abstract

An inhomogeneous magnetic exchange field at a superconductor/ferromagnet interface converts spin-singlet Cooper pairs to a spin-polarized triplet state. Although the decay envelope of triplet pairs within ferromagnetic materials is well studied, little is known about their decay in nonmagnetic metals and superconductors and, in particular, in the presence of spin-orbit coupling (SOC). Here, we investigate devices in which singlet and triplet supercurrents propagate into the s-wave superconductor Nb. In the normal state of Nb, triplet supercurrents decay over a distance of 5 nm, which is an order of magnitude smaller than the decay of spin-singlet pairs due to the SOC. In the superconducting state of Nb, triplet supercurrents are not able to couple with the singlet wave function and are thus blocked by the absence of available equilibrium states in the singlet gap. The results offer insight into the dynamics between s-wave singlet and s-wave triplet states.

## Teaser

Spin-polarized supercurrents interact with spin-orbit coupling and experience resistance in a superconductor.

## INTRODUCTION

Spin information can be transferred between ferromagnets through a superconducting spacer via spin-polarized quasi-particles or spin-polarized triplet Cooper pairs. Below the critical temperature of an s-wave superconductor, an energy gap opens in the density of states (DoS) below which the electrons form pairs with antiparallel spins in a singlet state, meaning singlet supercurrents do not carry a net spin. However, in this state, the spin relaxation time for spin-polarized quasi-particle (i.e., nonsuperconducting carrier) currents injected from a ferromagnet into a superconductor at the energy gap edge is enhanced by six orders of magnitude over the normal state (*1*, *2*). Spin can also be carried directly in the superconducting state through the conversion of singlet pairs into spin-polarized triplet pairs (*3*–*5*) at magnetically inhomogeneous superconductor/ferromagnet (S/F) interfaces via spin-mixing and spin-rotation processes (*3*–*10*). Spin-triplet Cooper pairs have a spin degree of freedom, and triplet supercurrents carry a net spin polarization. For s-wave spin-triplet pairs, the antisymmetric wave function under an overall exchange of fermions is maintained through the odd-frequency pairing state (*11*, *12*). The majority of experiments to detect triplet supercurrents are based on S/F_L_/F/F_R_/S devices (*9*) in which the magnetization directions of the F_L_ and F_R_ layers are noncollinear to the magnetization direction of the central F. Examples include Nb/Ni/Cu/Co/Ru/Co/Cu/Ni/Nb devices (*13*, *14*) in which the magnetization directions of the outer Ni layers are orthogonal to the magnetization of the Co/Ru/Co synthetic antiferromagnet and Nb/Cr/Fe/Cr/Nb devices (*15*) where a spin-glass layer at the Fe/Cr interface provides magnetic inhomogeneity (*15*–*18*). Recently, ferromagnetic resonance spin-pumping experiments in Pt/Nb/Py/Nb/Pt structures have shown evidence for superconducting pure spin currents. In these structures, the strong spin-orbit coupling (SOC) in Pt in conjunction with a proximity-induced ferromagnetic exchange field from Py creates a triplet DoS in superconducting Nb through which pure spin currents pumped from Py can propagate with a greater efficiency than when Nb is in the normal state (*19*, *20*).

Triplet pairs offer the potential for controlling spin and charge degrees of freedom via superconducting phase coherence (*3*, *4*, *21*–*23*); however, triplet device development requires an understanding of the decay envelope of generated triplet pairs in F, S, and N (nonmagnetic) metals (i.e., the coherence length of triplet pairs extracted from the source S), as well as an understanding of the dynamic interaction of singlet and triplet states.

Spin-mixing and spin-rotation at an interface or a magnetic exchange field with SOC (*19*, *20*, *24*, *25*) are required to transform singlet pairs into triplet pairs. Away from such an interface, the triplet pairs that are already formed should propagate through a second interface into an F, N, or S metal and transfer spin and the triplet wave function through these layers. In a ferromagnet, triplet pairs remain coherent over of tens of nanometers (*13*–*15*, *26*) and potentially hundreds of nanometers in half-metallic ferromagnets (*27*, *28*). Although little work has been done to explore triplet decay lengths in N metals, it is assumed that triplet pairs will remain coherent in N over the spin diffusion length (*6*, *13*). Hence, a notable difference in the proximity decay lengths of singlet and triplet pairs is expected in N metals since SOC will scatter the net spin carried by a triplet supercurrent (*6*, *25*) and not the charge carried by a zero net spin-singlet supercurrent.

A difference in the decay lengths is also expected for triplet and singlet pairs within an s-wave S. An attraction between electrons with opposite spin projections inside the s-wave superconductor supports the transfer of singlet pairs through the S layer without any damping. However, triplet pairs that penetrate a superconductor experience a spatial decay of their wave function since the singlet gap does not support electrons with equal spin projections.

Here, we investigate the triplet coherence in Nb, a metal with strong SOC (*29*–*31*). The triplet coherence length is investigated in both the normal and superconducting states by fabricating four series of S/F_L_/S′/F_R_/S devices: (A) “triplet control devices” Nb(300)/Cr(1)/Fe(*d*_Fe_)/Cr(1)/Nb(300) (thicknesses in nanometers) without S′ (also denoted as Nb′) and varying the total thickness of Fe from 3 to 15 nm to confirm singlet-to-triplet pair conversion at the Cr/Fe and Fe/Cr spin-mixer interfaces; (B) “singlet devices” Nb(300)/Cr(1)/Fe(2)/Nb′(*d*_Nb′_)/Fe(2)/Cr(1)/Nb(300) in which the total Fe thickness is low enough such that a residual singlet supercurrent is measurable; and two series of “triplet devices” with (C) Nb(300)/Cr(1)/Fe(4.8)/Nb′(*d*_Nb′_)/Fe(2.4)/Cr(1)/Nb(300) and (D) Nb(300)/Cr(1)/Fe(7.5)/Nb′(*d*_Nb′_)/Fe(2.0)/Cr(1)/Nb(300) layers with a total Fe thickness exceeding the maximum thickness for which a singlet supercurrent is observed in Nb/Fe/Nb devices (5.5 nm) (*15*). Each set of devices was prepared in a single deposition run. In device series (B) to (D), there are no intentional spin-mixing and spin-rotation interfaces between the Fe layers and the central Nb′ layer, and hence, a triplet pair wave function should not be generated in Nb′ in the superconducting state.

Current-perpendicular-to-plane S/F_L_/S′/F_R_/S Josephson devices are fabricated using a focused ion beam microscope technique that is described in detail elsewhere (*32*). Because of variations in the cross-sectional areas of the devices, the Josephson critical current (*I*_c_) is multiplied by the device normal state resistance *R*_n_ (estimated from the voltage at high current bias) to give the characteristic voltage (*I*_c_*R*_n_). The *I*_c_*R*_n_ of all devices is systematically investigated as a function of *d*_Nb′_ in the 0- to 40-nm range.

## RESULTS AND DISCUSSION

**Fig. 1:**
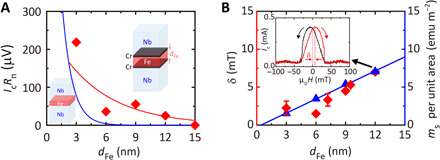
Triplet pair creation and magnetization properties of Fe/Cr interfaces. (**A**) The decay of the average critical current multiplied by the normal state resistance (*I*_c_*R*_n_) versus Fe thickness (*d*_Fe_) for Nb(300)/Cr(1)/Fe(*d*_Fe_)/Cr(1)/Nb(300) triplet control devices (red diamonds) at 1.6 K along with the known *d*_Fe_-decay of *I*_c_*R*_n_ for Nb(300)/Fe(*d*_Fe_)/Nb(300) (the blue curve) singlet devices with a coherence length of 1.0 nm (*15*). The (red) curve is a least square fit giving a triplet coherence length of ξ_F_^triplet^ = 5.3 ± 1.9 nm. (**B**) In-plane magnetic hysteresis (δ; red diamonds, left axis) estimated from the Nb(300)/Cr(1)/Fe(*d*_Fe_)/Cr(1)/Nb(300) triplet control devices at 1.6 K where δ is the maximum field shift in *I*_c_ (*H*). The right axis shows the magnetic moment at magnetic saturation per unit area (*m*_s_/m^2^) determined from unpatterned Nb(300)/Cr(1)/Fe(*d*_Fe_)/Cr(1)/Nb(300) films (blue triangles). The (blue) curve is a least-squares regression line fit to *m*_s_/m^2^ versus *d*_Fe_ with a volume magnetization of 618 electromagnetic unit (emu) cm^−3^ and a magnetically dead layer at each Fe/Cr interfaces of 0.2 to 0.3 nm. The inset shows an *I*_c_ (*H*) pattern for a Nb(300)/Cr(1)/Fe(12)/Cr(1)/Nb(300) device at 1.6 K.

We first discuss the triplet control devices. In Fig. 1A, we compare the *I*_c_*R*_n_ for these devices with Nb/Fe/Nb devices (blue curve) previously measured by our group (*15*) versus Fe layer thickness (*d*_Fe_) at 1.6 K. The Nb/Fe/Nb devices do not have (intentional) spin-mixer interfaces, and so, transport is spin-singlet. For *d*_Fe_ < 5 nm, supercurrents are detectable in both types of devices, but for *d*_Fe_ > 5 nm, supercurrents are only detectable in the triplet control devices confirming spin-mixing and spin-rotation at the Fe/Cr interfaces. The deviation from the exponential fit for the device with *d*_Fe_ = 6 nm is probably due to the sample-to-sample variation.

By applying a magnetic field (*H*) parallel to the interfaces, the *I*_c_ of the triplet control devices is modulated (inset of Fig. 1B). *I*_c_ (*H*) is hysteretic and the maximum values of *I*_c_ are obtained at nonzero applied field (μ_0_*H* = δ) due to the barrier magnetization. In Fig. 1B, we have plotted δ at 1.6 K (left axis) versus *d*_Fe_, which shows a linear increase in δ with *d*_Fe_, consistent with the linear rise in the magnetic moment (*m*_s_) per unit area with *d*_Fe_ for the unpatterned Nb/Cr/Fe/Cr/Nb films measured using a vibrating sample magnetometer at 300 K (right axis). Both δ and *m*_s_ per unit area are proportional to *d*_Fe_, suggesting that the Fe layers are homogeneously magnetized at magnetic saturation in both the unpatterned films and devices. From Fig. 1B, we estimate a magnetically dead layer at each Fe/Cr interfaces of 0.2 to 0.3 nm, which likely constitutes a spin glass (*15*–*18*).

**Fig. 2:**
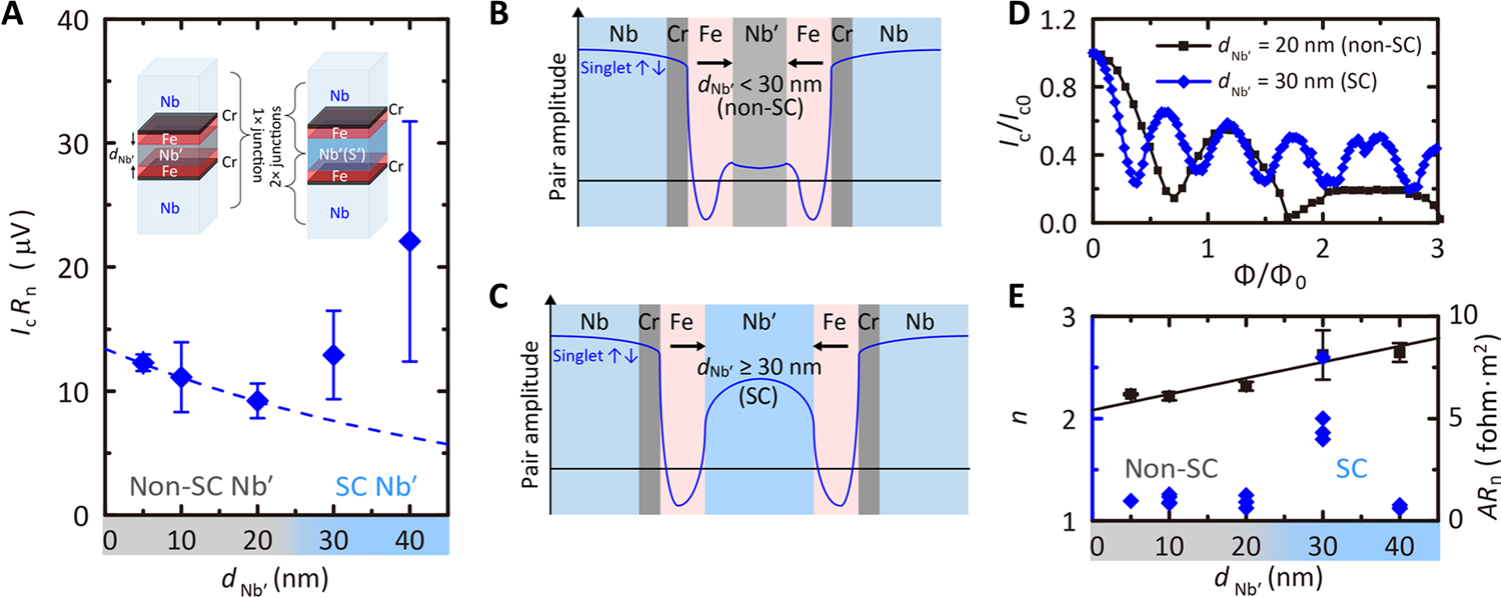
Supercurrents in singlet devices. (**A**) Characteristic voltage (*I*_c_*R*_n_) versus *d*_Nb′_ at 1.6 K for Nb(300)/Cr(1)/Fe(2)/Nb′(*d*_Nb′_)/Fe(2)/Cr(1)/Nb(300) devices. The dashed curve is a least square regression line fit to *I*_c_*R*_n_ for *d*_Nb′_ < 25 nm, where Nb’ is nonsuperconducting (non-SC), giving a singlet coherence length of ξ_N_^singlet^ = 52 ± 22 nm. The error bars in *I*_c_*R*_n_ represent the statistical average values of *I*_c_*R*_n_ for multiple devices at a given value of *d*_Nb′_, taking into account the errors in *I*_c_ and *R*_n_. (**B** and **C**) Schematic illustrations of the superconducting pair amplitudes for *d*_Nb′_ < 30 nm and *d*_Nb′_ ≥ 30 nm. (**D**) Normalized critical current versus normalized magnetic flux (Φ/Φ_0_) for Nb(300)/Cr(1)/Fe(2)/Nb′(20)/Fe(2)/Cr(1)/Nb(300) (black squares) and Nb(300)/Cr(1)/Fe(2)/Nb′(30)/Fe(2)/Cr(1)/Nb(300) (blue diamonds) devices at 1.6 K. (**E**) Normalized inverse periodicity of Fraunhofer oscillation (*n*; blue diamonds, left axis) and specific normal state resistance (*AR*_n_; black squares, right axis) versus *d*_Nb′_ for the singlet devices at 1.6 K with a least-squares regression line fit to *AR*_n_ (black line) from which we estimate ρ_Nb′_ ≈ 7.8 ± 1.1 μohm·cm. *R*_n_ values for *d*_Nb′_ = 30 and 40 nm are taken when Nb′ is in the normal state.

In Fig. 2A, we have plotted *I*_c_*R*_n_ versus *d*_Nb′_ for the singlet devices, which show two Nb′-thickness regimes: for *d*_Nb′_ < 30 nm, *I*_c_*R*_n_ slowly decreases with increasing *d*_Nb′_ and rises beyond 30 nm, indicating the onset of superconductivity in Nb′, which leads to two Josephson devices operating in series with the effective barrier thickness reduced as illustrated in Fig. 2 (B and C). Since the potential injection of spin-polarized quasi-particles suppresses the onset superconductivity of Nb′, it is difficult to distinguish the critical current of Nb′ and the Josephson critical current of the two devices. However, the formation of two Josephson devices in series is confirmed by a second harmonic Fraunhofer pattern, which results from the overlap of the Andreev bound states in Nb′ (*33*–*35*). In Fig. 2D, we have plotted the positive field direction in *I*_c_ (*H*) for two representative devices for two different values of *d*_Nb′_ (20 and 30 nm). *I*_c_ is modulated with magnetic flux [Φ = μ_0_*HL*(2λ + *d*)] according to sinc (*n*Φ/Φ_0_), but the periodicity (1/*n*) is halved (*n* = 2) for the 30-nm device, consistent with a second harmonic current-phase relationship. Here, *L* is the length of the junction perpendicular to the field, λ = 110 nm (*36*, *37*) is an estimate of the London penetration depth of Nb, *d* is the effective barrier thickness, and Φ_0_ is a flux quantum. In Fig. 2E (left axis), we have plotted *n* versus *d*_Nb′_, which shows *n* = 1 behavior for all thicknesses except for the 30-nm device (which matches the singlet coherence length). The *n* = 1 behavior (i.e., the first harmonic) for the *d*_Nb′_ = 40-nm devices is consistent with weakly overlapped Andreev bound states (*33*, *34*). To calculate *n*, we used *d* = *d*_Nb′_ + 2*d*_Cr_ (2 nm) + 2*d*_Fe_ (4 nm) for *d*_Nb′_ < 30 nm and *d* = *d*_Cr_ (1 nm) + *d*_Fe_ (2 nm) for *d*_Nb′_ ≥ 30 nm. The relatively large error of *n* for *d*_Nb′_ = 30 nm indicates the crossover between the conventional first harmonic and the unconventional second harmonic behavior. The nonzero *I*_c_ (*H*) minima for the *d*_Nb′_ = 30-nm device may be due to the nonuniform supercurrent mediated by the superconducting Nb′.

From the total specific resistance of these devices (*AR*_n_) versus *d*_Nb′_ (Fig. 2E; right axis) and fitting a least-squares regression line, we estimate a resistivity in Nb′ of ρ_Nb′_ ≈ 7.8 ± 1.1 μohm·cm (where *A* is the device cross-sectional area). The effective electron mean free path is *l* = *m*_e_ *v*_F_/*N*_d_ ρ_Nb_ *e*^2^ ≈ 11.2 ± 1.4 nm, where *m*_e_ ≈ 9.1 × 10^−31^ kg is the (effective) electron mass, *v*_F_ = 1.37 × 10^6^ m s^−1^ is the Fermi velocity of Nb (*38*), *N*_d_ = 5.56 × 10^28^ m^−3^ is the number density of conduction electrons in Nb (*38*), and *e* is the electric charge. The electron diffusivity is *D*_N_ = *v*_F_*l*/3 ≈ (5.1 ± 0.6) × 10^−3^ m^2^ s^−1^, which gives a singlet coherence length of ξ_N_^singlet^ = (ℏ*D*_N_/2π*k*_B_*T*)^1/2^ ≈ 61 ± 4 nm, consistent with the decay of *I*_c_*R*_n_ versus *d*_Nb′_ for *d*_Nb′_ < 30 nm.

**Fig. 3:**
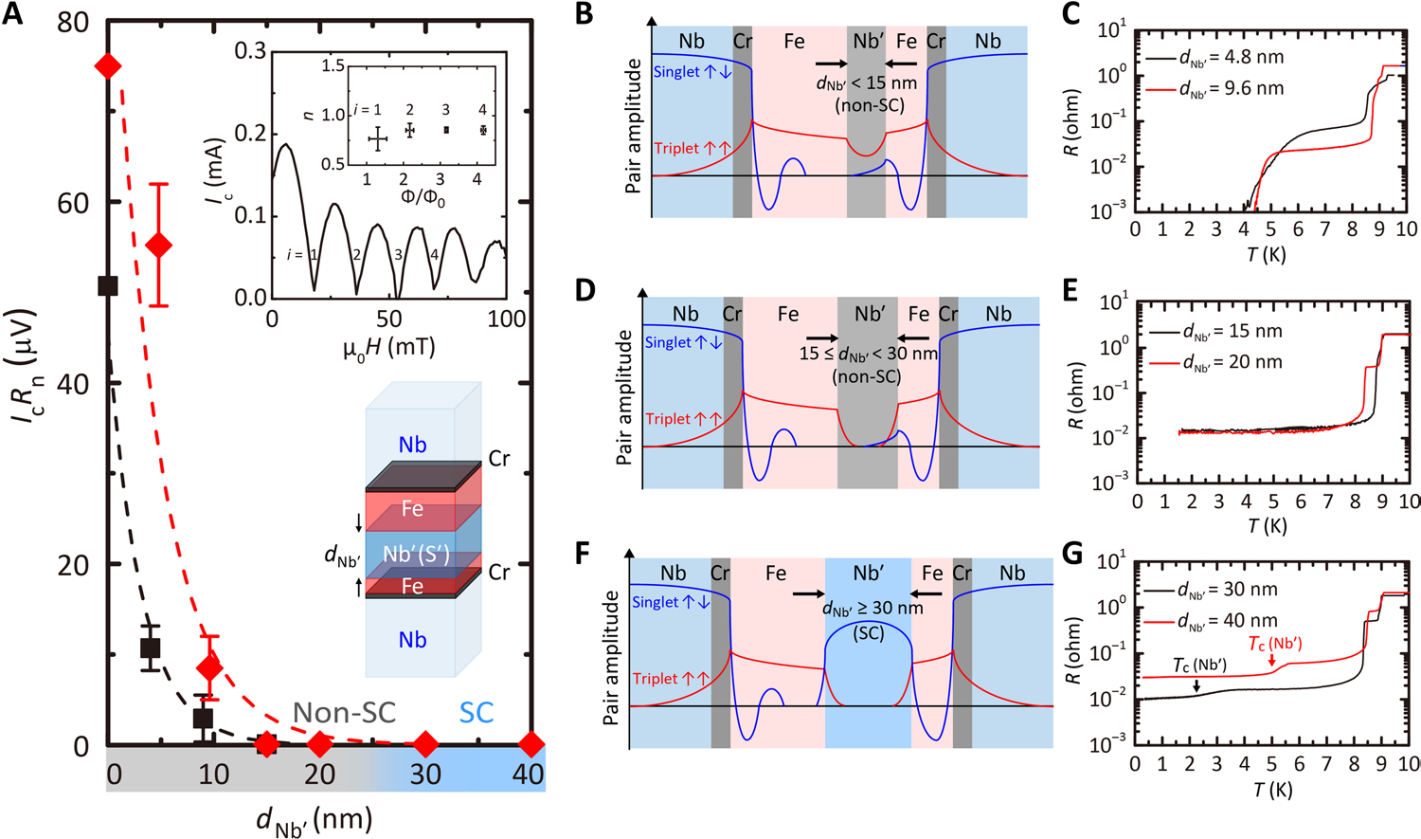
Supercurrents in triplet devices. (**A**) Characteristic voltage *I*_c_*R*_n_ versus thickness of the central Nb′ layer (*d*_Nb′_) at 1.6 K for Nb(300)/Cr(1)/Fe(4.8)/Nb′(*d*_Nb′_)/Fe(2.4)/Cr(1)/Nb(300) (red diamonds) and Nb(300)/Cr(1)/Fe(7.5)/Nb′(*d*_Nb′_)/Fe(2.0)/Cr(1)/Nb(300) (black squares) devices. The (red and black) dashed curves represent a least square regression line fit to *I*_c_*R*_n_, giving triplet coherence lengths of ξ_N_^triplet^ = 5.7 and 3.2 nm, respectively. The *I*_c_*R*_n_ values at *d*_Nb′_ = 0 nm are estimated from the gradient of *I*_c_*R*_n_ versus *d*_Fe_ for the Nb(300)/Cr(1)/Fe(*d*_Fe_)/Cr(1)/Nb(300) triplet devices in Fig. 1A. The error bars in *I*_c_*R*_n_ represent the statistical average values of *I*_c_*R*_n_ for multiple devices at a given value of *d*_Nb′_, taking into account the errors in *I*_c_ and *R*_n_. The inset shows an *I*_c_ (*H*) pattern for a Nb(300)/Cr(1)/Fe(7.5)/Nb′(4)/Fe(2.0)/Cr(1)/Nb(300) device at 1.6 K. (Top right of the inset) Distribution of intensity minimas in *I*_c_ (*H*) oscillations as a function of external magnetic flux for the triplet devices and corresponding normalized frequency (*n*) calculated by fitting *I*_c_ (*H*) to the standard Fraunhofer pattern (*39*). (**B** to **G**) Schematic illustrations of the superconducting pair amplitudes at 1.6 K and *R* (*T*) curves for *d*_Nb′_ < 15 nm, 15 ≤ *d*_Nb′_ < 30 nm, and *d*_Nb′_ ≥ 30 nm regimes in Nb(300)/Cr(1)/Fe(4.8)/Nb′(*d*_Nb′_)/Fe(2.4)/Cr(1)/Nb(300) devices.

The trend in *I*_c_*R*_n_ versus *d*_Nb′_ for both sets of triplet devices at 1.6 K is different from the singlet devices in that they do not show two-series junction behavior for all values of *d*_Nb′_ investigated (see Fig. 3). For *d*_Nb′_ < 15 nm, Josephson coupling is achieved (see Fig. 3B), and the corresponding resistance of the devices (*R*) falls to zero below 4 K. The inset of Fig. 3A shows an *I*_c_ (*H*) pattern for a Nb(300)/Cr(1)/Fe(7.5)/Nb′(4)/Fe(2.0)/Cr(1)/Nb(300) device showing standard Fraunhofer behavior with *I*_c_ shifted in field due to barrier flux from Fe. The periodicity of the Fraunhofer oscillation in the triplet devices is 77 to 86% of the first harmonic (*n* = 1) Fraunhofer pattern for a magnetized junction (*39*) (see top right of the inset in Fig. 3A). The slightly reduced *n* values and the slow decay of Fraunhofer oscillation may be due to the variation of the pair conversion efficiency within the device area.

Typical *R* (*T*) curves for *d*_Nb′_ < 15 nm are shown in Fig. 3C. The 300-nm-thick top and bottom Nb layers become superconducting below 9 K, showing a drop in *R* with the resistance continuously decreasing with decreasing temperature as superconductivity gradually proximitizes the Cr/Fe/Nb′/Fe/Cr barrier. The barriers are completely proximitized (*R* = 0) below 4 K. The decay in *I*_c_*R*_n_ versus *d*_Nb′_ is exponential [*I*_c_*R*_n_ = exp(−ξ_N_^triplet^/*d*_Nb′_)] with a triplet coherence length of ≈3.2 to 5.7 nm, which is an order of magnitude smaller than the singlet coherence length in Nb′ estimated from Fig. 2A. The strong pair breaking effect on triplet pairs is likely due to strong SOC in normal state Nb (*29*–*31*), which suppresses the triplet pairing coherence due to scattering of the spin associated with the triplet supercurrent (*6*, *25*). We note that, for all temperatures, we do not observe magnetoresistance from the Fe/Nb′/Fe barriers in these devices, suggesting a short spin diffusion length in thin Nb′ layers (<10 nm) in these particular devices due to SOC (*29*–*31*) (see the Supplementary Materials for details).

In the *d*_Nb′_ range of 15 to 30 nm, *R* of the devices does not fall to zero (Fig. 3E) and Josephson coupling is not detected (i.e., no *I*_c_), suggesting the absence of triplet or singlet supercurrents, i.e., the triplet pair amplitude across Nb′ is (approximately) zero. For *d*_Nb′_ ≥ 30 nm, the Nb′ spacers show a superconducting transition with dips in *R* below 2.3 and 5.0 K for *d*_Nb′_ = 30 and 40 nm, respectively (Fig. 3G). The resistivity of the Nb′ layer calculated from the resistance drop associated with the superconducting transition for these devices is 8.2 to 10.4 μohm·cm, consistent with the value estimated from Fig. 2E. In contrast to the singlet devices (Fig. 2A), we do not observe two-series junction behavior in which the superconducting Nb′ layer effectively halves the thickness of the barrier layers and leads to a higher *I*_c_*R*_n_ over the normal state Nb′, meaning that the triplet wave function is unable to mediate Josephson coupling with the singlet wave function of Nb′. The triplet supercurrent is blocked even for the device with the thinnest superconducting Nb′ layer (*d*_Nb′_ = 30 nm) obtained in this work, and hence, we estimate the coherence length of triplet pairs to be shorter than the singlet pair correlation length (≈30 nm). The disconnection of the triplet pair amplitude across the Nb′ layer blocks charge transport via triplet pairs, i.e., Nb′ is an effective insulator for triplet pairs.

In a related experiment, we investigated the superconducting DoS on NbN/La_2/3_Ca_1/3_MnO_3_ using scanning tunneling microscopy (*40*), where NbN is an s-wave superconductor and La_2/3_Ca_1/3_MnO_3_ is a highly spin-polarized ferromagnetic manganite. Here, an enhancement of the superconducting DoS in NbN was observed around zero energy, consistent with spin-one triplet theory assuming a magnetically inhomogeneous interface (*41*). In agreement with the present manuscript, the zero energy enhancement of the DoS in NbN rapidly decayed as a function of NbN thickness with a decay envelope close to the spin diffusion and superconducting coherence lengths; these results demonstrated that the proximity-induced triplet state in NbN was unfavorable within an intrinsic singlet DoS.

The differences in the coherence lengths of singlet and triplet pairs observed in F (Fe), N (normal state Nb′), and S′ (superconducting Nb′) are summarized in Table 1 together with the mean free path and the spin diffusion lengths. In F, the coherence length of triplet pairs is long-ranged and close to the spin diffusion length, while singlet pairs affected by the exchange field are short-ranged (Fig. 1A). In N with strong SOC, the coherence length of the triplet pairs is short-ranged (Fig. 3A) due to the short spin diffusion length (see the Supplementary Materials), while singlet pairs are unaffected by SOC and are long-ranged (Fig. 2A). In S′, singlet pairs couple with the singlet wave function of S′ and create two-series junction behavior, and hence, singlet supercurrents do not show a decay (Fig. 2A). Triplet pairs, however, are not able to couple with the singlet wave function of S′ and hence decay within the order of the singlet coherence length (30 nm; Fig. 3A).

**Table 1:** Electron mean free path (*l*), spin diffusion length (*l*_sd_), and coherence lengths (ξ) in Fe and Nb at 1.6 K.

**Length scale** **(nm)**	**Fe**	**Nb′ (non-SC)**	**Nb′ (SC)**
*l*	10.4 (*43*)	11.2 ± 1.4	–
*l*_sd_	8.5 ± 1.5 (*43*)	<4.8	–
ξ^singlet^	1.0 (*15*)	52 ± 22	No decay
ξ^triplet^	5.3 ± 1.9	4.5 ± 1.3	<30

Triplet pairs that are not able to couple with the singlet wave function can be blocked in the singlet superconducting Nb′ through SOC or (and) a competition with the singlet pairing correlation. There is no existing theory to explain the effect of SOC on triplet pairs in a material with a singlet pairing correlation. Assuming that the singlet pairing correlation of Nb′ does not affect the SOC scattering of triplet pairs and there is no interaction between the singlet and the triplet pairing states, the decay length of the triplet pairs in the superconducting Nb′ is obtained from the equation (5.36) in (*6*)

$$\xi_{S}^{\text{triplet}}\approx {\left\{2(\frac{4}{\tau_{\text{SO}}D_{N}})\right\}}^{-\frac{1}{2}}=\frac{1}{2}{\left(\frac{l_{\text{so}}l}{6}\right)}^{\frac{1}{2}}\approx 0.2\xi_{N}^{\text{singlet}}{\left(\frac{l_{\text{so}}}{\xi_{0}}\right)}^{\frac{1}{2}}\;\text{if}\;\frac{4\hbar }{\tau_{\text{SO}}}\gg k_{B}T_{c}$$ (1)

where ξ_0_ = 0.18ℏ*v*_F_/*k*_B_*T*_c_, and *l*_so_ and τ_so_ are the mean free length and the mean free time between the spin-orbit scattering events, respectively. A rough estimation *l*_so_ ≈ *l*_sd_ ≈ 5 nm, ξ_0_ ≈ 30 nm, and ξ_N_^singlet^ ≈ 52 ± 22 nm gives ξ_S_^triplet^ ≈ 4.2 ± 1.8 nm, consistent with the experimental results showing a blocking of triplet supercurrents in a singlet superconducting Nb′ (ξ_S_^triplet^ < 30 nm) and matching with ξ_N_^triplet^ ≈ 4.5 ± 1.3 nm estimated from Fig. 3A.

However, in the presence of the singlet pairing correlation, triplet pairs would no longer experience an effective field due to the SOC since the condensate requires a matching DoS for up and down spin electrons—hence, superconductivity and a supercurrent are immune to the presence of SOC. If this is the case, the strong suppression of triplet pairs is dominated by a competition between the singlet and the triplet pairing states (*42*) resulting from the fact that they have an opposite influence on the electron DoS at the Fermi energy, i.e., the singlet pairing decreases it, while the triplet correlations lead to its increase. To show the effect of singlet pairing correlation on the decay of triplet pairs, we calculate the critical current density in a S_L_/F_L_/S′/F_R_/S_R_ device where S_L_/F_L_ and F_R_/S_R_ are spin-mixing/rotation interfaces and each layer is atomically thin. The central S′ layer has a superconducting gap of Δ_0_, which is smaller than that of S_L_ and S_R_ (Δ_1_). The magnetic exchange fields of F_L_ and F_R_ layers (spin-rotation axis) are parallel to each other and strong enough to block the transport of minority spin triplet pairs. By solving the Gor’kov equations derived from a hopping probability of electrons between the atomically thin layers (see the Supplementary Materials for details), we obtain the critical current density that appears to be completely triplet

$$J_{c}={|\Delta_{1}|}^{2}h_{L}h_{R}\text{sin}\theta_{L}\text{sin}\theta_{R}(a-b{|\Delta_{0}|}^{2})$$ (2)

where *h*_L_ (*h*_R_) is the magnetic exchange field in F_L_ (F_R_), and θ_L_ (θ_R_) is the magnetization angle between the magnetic exchange field at the S_L_/F_L_ (F_R_/S_R_) interface and F_L_ (F_R_). We note that Eq. 2 obtained from the anomalous Green’s functions in S′ consists of only triplet supercurrents and a singlet component is absent, meaning that phase-coupling between triplet pairs and the singlet wave function in S′ is not mediated, agreeing with the experimental results. Since the coefficients *a*, *b* > 0, the presence of a singlet gap in S′ layer (Δ_0_) suppresses the triplet current density. This results from the fact that Δ_0_ suppresses the triplet component of the anomalous Green’s function (i.e., the motion of triplet pairs), which also agrees with the decay of triplet pairs within the length scale of singlet coherence length shown in Fig. 3.

We have observed a strong suppression of spin-triplet supercurrents in the normal and superconducting states of the s-wave superconductor Nb. In the normal state, SOC rapidly scatters triplet pairs, and in the superconducting state, triplet pairs are not able to mediate phase-coupling and are blocked, qualitatively consistent with our theoretical model. Although the exact underlying mechanism(s) for triplet pair suppression in an s-wave gap remains an open question, the results provide insight into the dynamic coupling of s-wave singlet and s-wave triplet states demonstrating a mechanism for superconducting filtering of triplet pairs.

## MATERIALS AND METHODS

### Film growth

Unpatterned films were fabricated on 5 mm × 5 mm quartz substrates by direct current magnetron sputtering in an ultrahigh-vacuum chamber with a base pressure better than 10^−6^ Pa. The sputtering targets were presputtered for approximately 20 min to clean the surfaces, and the films were grown using an Ar pressure of 1.5 Pa. Multiple quartz substrates were placed on a rotating circular table that passed in series under stationary magnetrons so that multiple samples with different layer thicknesses could be grown in the same deposition run. The thickness of each layer was controlled by adjusting the angular speed of the rotating table at which the substrates moved under the respective targets and the sputtering power.

### Device fabrication

Standard optical lithography and Ar-ion milling define 4-μm-wide tracks, which were narrowed using a focused beam of Ga ions (Zeiss Crossbeam 540) to make current-perpendicular-to-plane devices. Further details on the device fabrication process are described elsewhere (*32*). A typical device dimension is 500 nm by 500 nm.

### Transport measurements

A pulse tube cryogen-free system (Cryogenic Ltd.) was used to cool the devices down to 1.6 K. Resistivity and current-voltage *I* (*V*) characteristics of the devices were measured in a four-point configuration using a current-bias circuit attached to a lock-in amplifier and an analog-digital converter and also using the differential conductance mode of a Keithley 6221 AC current source and a 2182A nanovoltmeter. The Josephson critical current *I*_c_ and the normal state resistance *R*_n_ of a device were determined by fitting the *I* (*V*) characteristics to the resistively shunted junction model *V* = *R*_n_ (*I*^2^ − *I*_c_^2^)^0.5^.

## Supplementary Material

http://advances.sciencemag.org/cgi/content/full/7/3/eabe0128/DC1

Adobe PDF - abe0128_SM.pdf

Spin-orbit coupling suppresion and singlet-state blocking of spin-triplet Cooper pairs

## SUPPLEMENTARY MATERIALS

Supplementary material for this article is available at http://advances.sciencemag.org/cgi/content/full/7/3/eabe0128/DC1
