# Supplementary material for: Spin-orbit coupling suppression and singlet-state blocking of spin-triplet Cooper pairs
Source: Sci Adv. 2021 Jan 13;7(3):eabe0128. doi: 10.1126/sciadv.abe0128 (PMC7806214; doi:10.1126/sciadv.abe0128)
Supplement: http://advances.sciencemag.org/cgi/content/full/7/3/eabe0128/DC1 [file supp_7_3_eabe0128__index.html]

Science Advances | Science AdvancesAAASSearchScience AdvancesMenu

## Supplementary Materials

# Spin-orbit coupling suppression and singlet-state blocking of spin-triplet Cooper pairs

Sachio Komori, James M. Devine-Stoneman, Kohei Ohnishi, Guang Yang, Zhanna Devizorova, Sergey Mironov, Xavier Montiel, Linde A. B. Olde Olthof, Lesley F. Cohen, Hidekazu Kurebayashi, Mark G. Blamire, Alexandre I. Buzdin, Jason W. A. Robinson

Download Supplement

**This PDF file includes:**

- Sections S1 and S2
- Figs. S1 and S2

**Files in this Data Supplement:**

- Adobe PDF - abe0128\_SM.pdf
