# Supplementary material for: Spin-orbit coupling suppression and singlet-state blocking of spin-triplet Cooper pairs
Source: Sci Adv. 2021 Jan 13;7(3):eabe0128. doi: 10.1126/sciadv.abe0128 (PMC7806214; doi:10.1126/sciadv.abe0128)
Supplement: http://advances.sciencemag.org/cgi/content/full/7/3/eabe0128/DC1 [file supp_7_3_eabe0128__1.pdf]

Science Advances
AAAS

advances.sciencemag.org/cgi/content/full/7/3/eabe0128/DC1

Supplementary Materials for

Spin-orbit coupling suppression and singlet-state blocking of spin-triplet Cooper pairs

Sachio Komori*, James M. Devine-Stoneman, Kohei Ohnishi, Guang Yang, Zhanna Devizorova, Sergey Mironov, Xavier Montiel, Linde A. B. Olde Olthof, Lesley F. Cohen, Hidekazu Kurebayashi, Mark G. Blamire, Alexandre I. Buzdin, Jason W. A. Robinson*

*Corresponding author. Email: sk891@cam.ac.uk (S.K.); jjr33@cam.ac.uk (J.W.A.R.)

Published 13 January 2021, *Sci. Adv.* **7**, eabe0128 (2021)
DOI: 10.1126/sciadv.abe0128

This PDF file includes:

Sections S1 and S2
Figs. S1 and S2

Section S1: Spin-transport in normal state Nb

Figure S1 shows the electrical resistance (R) versus an in-plane magnetic field (H) for Nb(300 nm)/Cr(1 nm)/Fe(4.8 nm)/Cu(10 nm)/Fe(2.4 nm)/Cr(1 nm)/Nb(300 nm) (left-axis) and Nb(300 nm)/Cr(1 nm)/Fe(4.8 nm)/Nb(4.8 nm)/Fe(2.4 nm)/Cr(1 nm)/Nb(300 nm) (right-axis) devices at 10 K. A mismatch between the coercive fields of the 4.8-nm-thick Fe layer and the 2.4-nm-thick Fe layer leads to an increase in R at $\mu_0 H \approx 50$ mT due to giant magnetoresistance (GMR) effect in the device with a 10-nm-thick Cu spacer but not in the device with a 4.8-nm-thick Nb spacer. The current is applied perpendicular to the plane in the device and hence the thickness of the spacer should be less than the spin-diffusion length to observe GMR. The absence of GMR in the device with a Nb spacer indicates a short spin-diffusion length (< 5 nm) in the normal state of Nb in these devices which agrees with the decay envelope of triplet supercurrents in Fig. 3A in the main text.

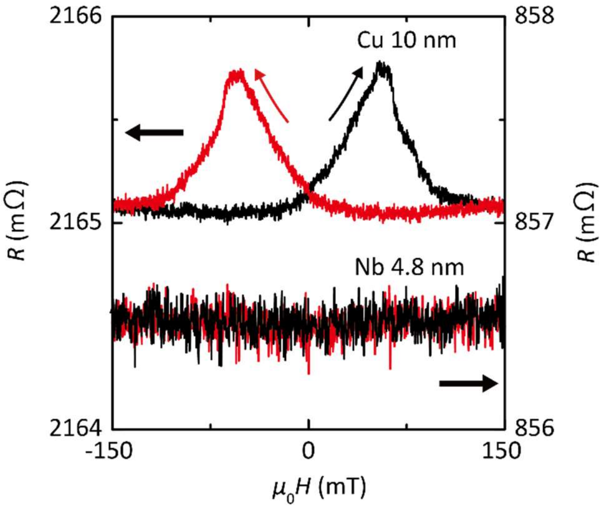

Fig. S1. ***R*** **(*H*) for Nb(300)/Cr(1)/Fe(4.8)/Cu(10)/Fe(2.4)/Cr(1)/Nb(300) (left-axis) and Nb(300)/Cr(1)/Fe(4.8)/Nb(4.8)/Fe(2.4)/Cr(1)/Nb(300) (right-axis) devices at *T* = 10 K.**

Section S2: Theory of the suppression of spin-triplet Josephson currents in a singlet superconductor

We consider a $S_1/F_1/S'/F_2/S_2$ Josephson junction (see Fig. S2) consisting of atomically thin superconductors (S_1 and S_2), ferromagnets (F_1 and F_2) and a central superconductor (S'). The neighbouring layers are coupled by the transfer integrals t_i, ($i = 1, 2, 3, 4$) of the tight-binding model. The critical temperature of the superconducting leads S_1 and S_2 ($T_{c1} = T_{c2}$) is higher than that of the central superconductor S' (T_{c0}). Thus, the central layer S' can be both in the normal and in the superconducting states at $T < T_{c1}$. We assume that $T \approx T_{c0} < T_{c1}$, $t_i << T_{c0}$ and the interlayer tunneling conserves the momentum. Also, we assume that the S_1/F_1 and F_2/S_2 interfaces are magnetized. The misalignment angle θ_i ($i = 1, 2$) between the exchange field $\mathbf{h}_i = h_i(\cos\theta_i\,\mathbf{z} + \sin\theta_i\,\mathbf{x})$ at the S_i/F_i interface and the spin-rotation axis $\mathbf{z}$ in the F_i layer gives rise to the emergence of the spin-triplet superconducting correlations. We assume 100 % spin polarization of F_1 and F_2 layers and therefore the transport of minority spin triplet pairs is blocked.

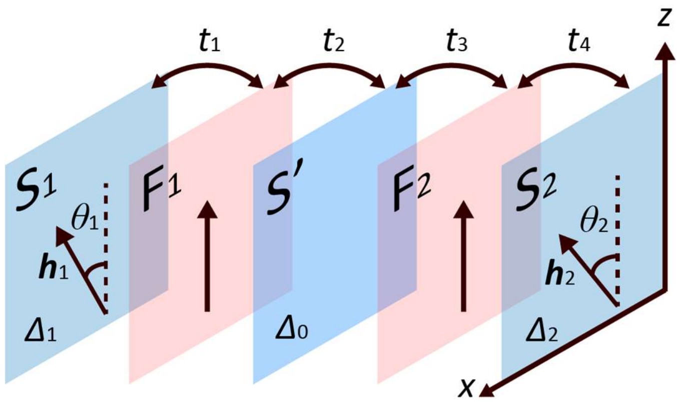

Fig. S2. $S_1/F_1/S'/F_2/S_2$ Josephson junction consisting of atomically thin layers.

The superconducting gaps in S', S_1 and S_2 layers are Δ_0, $\Delta_1 = |\Delta_1|e^{-i\varphi/2}$ and $\Delta_2 = |\Delta_2|e^{i\varphi/2}$ with $|\Delta_1| = |\Delta_2|$ (the phase difference across the junction equals to φ). The energy spectrum in the superconductors is $\xi(\mathbf{p})$, while in the ferromagnets it is spin-dependent: $\xi_\uparrow = \xi(\mathbf{p})$ and $\xi_\downarrow = +\infty$. We denote the electron annihilation operators in S_1, F_1, S', F_2, S_2 layers as $\hat{\eta}$, $\hat{\psi}$, $\hat{\phi}$, $\hat{\hat{\psi}}$ and $\hat{\hat{\eta}}$. The Hamiltonian in the system under consideration is

$$\hat{H} = \hat{H}_0 + \hat{H}_{\text{BCS}} + \hat{H}_t, \tag{S1}$$

with $\hat{H}_0$ the single particle Hamiltonian describing the kinetic energy in the five layers. $\hat{H}_0$ writes:

$$\hat{H}_0 = \sum_{\mathbf{p};\alpha,\beta=\uparrow,\downarrow} \xi(\mathbf{p})\,\hat{\phi}^\dagger_{\mathbf{p},\alpha}\hat{\phi}_{\mathbf{p},\beta}\delta_{\alpha\beta} + \hat{P}_{\alpha\beta}\hat{\psi}^\dagger_{\mathbf{p},\alpha}\hat{\psi}_{\mathbf{p},\beta} + \hat{P}_{\alpha\beta}\hat{\hat{\psi}}^\dagger_{\mathbf{p},\alpha}\hat{\hat{\psi}}_{\mathbf{p},\beta} + \hat{C}^{(1)}_{\alpha\beta}\hat{\eta}^\dagger_{\mathbf{p},\alpha}\hat{\eta}_{\mathbf{p},\beta}$$
$$+ \hat{C}^{(2)}_{\alpha\beta}\hat{\hat{\eta}}^\dagger_{\mathbf{p},\alpha}\hat{\hat{\eta}}_{\mathbf{p},\beta} \tag{S2}$$

where the coefficient $\hat{C}_i$ ($i = 1,2$) describes the modified kinetic energy due to the magnetized interfaces and $\hat{P}$ is the spin-dependent kinetic energy of the ferromagnets (assumed identical for F_1 and F_2), given by

$$\hat{C}^{(i)} = \begin{pmatrix} \xi - h_i\cos\theta_i & -h_i\sin\theta_i \\ -h_i\sin\theta_i & \xi + h_i\cos\theta_i \end{pmatrix}, \qquad \hat{P} = \begin{pmatrix} \xi_\uparrow & 0 \\ 0 & \xi_\downarrow \end{pmatrix} = \begin{pmatrix} \xi & 0 \\ 0 & \infty \end{pmatrix}.$$

The second term in (S1), $\hat{H}_{\text{BCS}}$ describes the superconductivity in the three superconductors as

$$\hat{H}_{\text{BCS}} = \sum_{\mathbf{p}} \Delta_0\hat{\Phi}^\dagger_{\mathbf{p},\uparrow}\hat{\Phi}^\dagger_{-\mathbf{p},\downarrow} + \Delta_0^*\hat{\Phi}_{-\mathbf{p},\downarrow}\hat{\Phi}_{\mathbf{p},\uparrow} + \Delta_1\hat{\eta}^\dagger_{\mathbf{p},\uparrow}\hat{\eta}^\dagger_{-\mathbf{p},\downarrow} + \Delta_1^*\hat{\eta}_{-\mathbf{p},\downarrow}\hat{\eta}_{\mathbf{p},\uparrow} + \Delta_2\hat{\hat{\eta}}^\dagger_{\mathbf{p},\uparrow}\hat{\hat{\eta}}^\dagger_{-\mathbf{p},\downarrow}$$
$$+ \Delta_2^*\hat{\hat{\eta}}_{-\mathbf{p},\downarrow}\hat{\hat{\eta}}_{\mathbf{p},\uparrow} \tag{S3}$$

and $\hat{H}_t$ is the tunnelling Hamiltonian given by

$$
\begin{aligned}
\hat{H}_t = \sum_{\mathbf{p};\alpha} t_1 \left(\eta^{\dagger}_{\mathbf{p},\alpha}\psi_{\mathbf{p},\alpha} + \psi^{\dagger}_{\mathbf{p},\alpha}\eta_{\mathbf{p},\alpha}\right) + t_2\left(\psi^{\dagger}_{\mathbf{p},\alpha}\phi_{\mathbf{p},\alpha} + \phi^{\dagger}_{\mathbf{p},\alpha}\psi_{\mathbf{p},\alpha}\right) + t_3\left(\phi^{\dagger}_{\mathbf{p},\alpha}\tilde{\psi}_{\mathbf{p},\alpha} + \tilde{\psi}^{\dagger}_{\mathbf{p},\alpha}\phi_{\mathbf{p},\alpha}\right) \\
+ t_4\left(\tilde{\psi}^{\dagger}_{\mathbf{p},\alpha}\tilde{\eta}_{\mathbf{p},\alpha} + \tilde{\eta}^{\dagger}_{\mathbf{p},\alpha}\tilde{\psi}_{\mathbf{p},\alpha}\right). \qquad \text{(S4)}
\end{aligned}
$$

The commutation relations with the Hamiltonian are

$$
\begin{aligned}
\left[\hat{H},\hat{\phi}_{\mathbf{p},\uparrow}\right] &= -\xi\hat{\phi}_{\mathbf{p},\uparrow} + \Delta_0\hat{\phi}^{\dagger}_{-\mathbf{p},\downarrow} - t_2\hat{\psi}_{\mathbf{p},\uparrow} - t_3\hat{\tilde{\psi}}_{\mathbf{p},\uparrow}, \\
\left[\hat{H},\hat{\phi}^{\dagger}_{-\mathbf{p},\downarrow}\right] &= \xi\hat{\phi}^{\dagger}_{-\mathbf{p},\downarrow} + \Delta_0^*\hat{\phi}_{\mathbf{p},\uparrow} + t_2\hat{\psi}^{\dagger}_{-\mathbf{p},\downarrow} + t_3\hat{\tilde{\psi}}^{\dagger}_{-\mathbf{p},\downarrow}, \\
\left[\hat{H},\hat{\psi}_{\mathbf{p},\uparrow}\right] &= -\sum_{\beta} P_{\uparrow\beta}\hat{\psi}_{\mathbf{p},\beta} - t_1\hat{\eta}_{\mathbf{p},\uparrow} - t_2\hat{\phi}_{\mathbf{p},\uparrow}, \\
\left[\hat{H},\hat{\psi}^{\dagger}_{-\mathbf{p},\downarrow}\right] &= \sum_{\alpha} P_{\alpha\downarrow}\hat{\psi}^{\dagger}_{-\mathbf{p},\alpha} + t_1\hat{\eta}^{\dagger}_{-\mathbf{p},\downarrow} + t_2\hat{\phi}^{\dagger}_{-\mathbf{p},\downarrow}, \\
\left[\hat{H},\hat{\tilde{\psi}}_{\mathbf{p},\uparrow}\right] &= -\sum_{\beta} P_{\uparrow\beta}\hat{\tilde{\psi}}_{\mathbf{p},\beta} - t_3\hat{\phi}_{\mathbf{p},\uparrow} - t_4\hat{\tilde{\eta}}_{\mathbf{p},\uparrow}, \\
\left[\hat{H},\hat{\tilde{\psi}}^{\dagger}_{-\mathbf{p},\downarrow}\right] &= \sum_{\alpha} P_{\alpha\downarrow}\hat{\tilde{\psi}}^{\dagger}_{-\mathbf{p},\alpha} + t_3\hat{\phi}^{\dagger}_{-\mathbf{p},\downarrow} + t_4\hat{\tilde{\eta}}^{\dagger}_{-\mathbf{p},\downarrow}, \\
\left[\hat{H},\tilde{\eta}_{\mathbf{p},\uparrow}\right] &= -\sum_{\beta} \hat{C}^{(1)}_{\uparrow\beta}\hat{\eta}_{\mathbf{p},\beta} + \Delta_1\hat{\eta}^{\dagger}_{-\mathbf{p},\downarrow} - t_1\hat{\psi}_{\mathbf{p},\uparrow}, \\
\left[\hat{H},\hat{\eta}^{\dagger}_{-\mathbf{p},\downarrow}\right] &= \sum_{\alpha} \hat{C}^{(1)}_{\alpha\downarrow}\hat{\eta}^{\dagger}_{-\mathbf{p},\alpha} + \Delta_1^*\hat{\eta}^{\dagger}_{\mathbf{p},\uparrow} + t_1\hat{\psi}^{\dagger}_{-\mathbf{p},\downarrow}, \\
\left[\hat{H},\hat{\tilde{\eta}}_{\mathbf{p},\uparrow}\right] &= -\sum_{\beta} \hat{C}^{(2)}_{\uparrow\beta}\hat{\tilde{\eta}}_{\mathbf{p},\beta} + \Delta_2\hat{\tilde{\eta}}^{\dagger}_{-\mathbf{p},\downarrow} - t_4\hat{\tilde{\psi}}_{\mathbf{p},\uparrow}, \\
\left[\hat{H},\hat{\tilde{\eta}}^{\dagger}_{-\mathbf{p},\downarrow}\right] &= \sum_{\alpha} \hat{C}^{(2)}_{\alpha\downarrow}\hat{\tilde{\eta}}^{\dagger}_{-\mathbf{p},\beta} + \Delta_2^*\hat{\tilde{\eta}}^{\dagger}_{\mathbf{p},\uparrow} + t_4\hat{\tilde{\psi}}^{\dagger}_{-\mathbf{p},\downarrow}.
\end{aligned}
$$

We assume the coherent electron tunneling between the layers, which preserves the in-plane momentum **p**. Using the Liouville equation

$$
i\frac{\partial\Psi}{\partial\tau} = \left[\hat{H},\Psi\right]
$$

we introduce the following Green's functions in the imaginary-time representation:

$$
\begin{aligned}
G_{\alpha\beta}(\mathbf{p};\tau_1,\tau_2) &= -\langle T_\tau\hat{\phi}_{\mathbf{p},\alpha}(\tau_1)\hat{\phi}^{\dagger}_{\mathbf{p},\beta}(\tau_2)\rangle, \quad & F^{\dagger}_{\alpha\beta}(\mathbf{p};\tau_1,\tau_2) &= \langle T_\tau\hat{\phi}^{\dagger}_{-\mathbf{p},\alpha}(\tau_1)\hat{\phi}^{\dagger}_{\mathbf{p},\beta}(\tau_2)\rangle, \\
E^{\psi}_{\alpha\beta}(\mathbf{p};\tau_1,\tau_2) &= -\langle T_\tau\hat{\psi}_{\mathbf{p},\alpha}(\tau_1)\hat{\phi}^{\dagger}_{\mathbf{p},\beta}(\tau_2)\rangle, \quad & F^{\psi\dagger}_{\alpha\beta}(\mathbf{p};\tau_1,\tau_2) &= \langle T_\tau\hat{\psi}^{\dagger}_{-\mathbf{p},\alpha}(\tau_1)\hat{\phi}^{\dagger}_{\mathbf{p},\beta}(\tau_2)\rangle, \\
E^{\tilde{\psi}}_{\alpha\beta}(\mathbf{p};\tau_1,\tau_2) &= -\langle T_\tau\hat{\tilde{\psi}}_{\mathbf{p},\alpha}(\tau_1)\hat{\phi}^{\dagger}_{\mathbf{p},\beta}(\tau_2)\rangle, \quad & F^{\tilde{\psi}\dagger}_{\alpha\beta}(\mathbf{p};\tau_1,\tau_2) &= \langle T_\tau\hat{\tilde{\psi}}^{\dagger}_{-\mathbf{p},\alpha}(\tau_1)\hat{\phi}^{\dagger}_{\mathbf{p},\beta}(\tau_2)\rangle, \\
E^{\eta}_{\alpha\beta}(\mathbf{p};\tau_1,\tau_2) &= -\langle T_\tau\hat{\eta}_{\mathbf{p},\alpha}(\tau_1)\hat{\phi}^{\dagger}_{\mathbf{p},\beta}(\tau_2)\rangle, \quad & F^{\eta\dagger}_{\alpha\beta}(\mathbf{p};\tau_1,\tau_2) &= \langle T_\tau\hat{\eta}^{\dagger}_{-\mathbf{p},\alpha}(\tau_1)\hat{\phi}^{\dagger}_{\mathbf{p},\beta}(\tau_2)\rangle, \\
E^{\tilde{\eta}}_{\alpha\beta}(\mathbf{p};\tau_1,\tau_2) &= -\langle T_\tau\hat{\tilde{\eta}}_{\mathbf{p},\alpha}(\tau_1)\hat{\phi}^{\dagger}_{\mathbf{p},\beta}(\tau_2)\rangle, \quad & F^{\tilde{\eta}\dagger}_{\alpha\beta}(\mathbf{p};\tau_1,\tau_2) &= \langle T_\tau\hat{\tilde{\eta}}^{\dagger}_{-\mathbf{p},\alpha}(\tau_1)\hat{\phi}^{\dagger}_{\mathbf{p},\beta}(\tau_2)\rangle,
\end{aligned}
$$

where G and $F^{\dagger}$ are the single-particle and anomalous Green's function in S', respectively. The tunneling Green's functions E^{Ψ}, $E^{\widetilde{\Psi}}$, E^{η} and $E^{\widetilde{\eta}}$ represent the tunneling of a particle from S' to F_1, F_2, S_1 and S_2, respectively. Finally, the anomalous Green's functions $F^{\Psi\dagger}$, $F^{\widetilde{\Psi}\dagger}$, $F^{\eta\dagger}$ and $F^{\widetilde{\eta}\dagger}$ are associated with the creation of a Cooper pair in which one electron is located in S′ and the other electron is in F_1, F_2, S_1 and S_2, respectively.

Rewriting the commutation relations in terms of the Green's functions and applying the Fourier transform such that $i\partial\Psi/\partial\tau = -i\omega\Psi$, we find the following closed set of matrices Gor'kov equations in frequency representation:

$$\text{for S':}\quad \begin{cases} (i\omega - \xi)G + i\Delta_0\sigma_y F^{\dagger} - t_2 I E^{\Psi} - t_3 I E^{\widetilde{\Psi}} = I, \\ (i\omega + \xi)F^{\dagger} - i\Delta_0^{*}\sigma_y G + t_2 I F^{\Psi\dagger} + t_3 I F^{\widetilde{\Psi}\dagger} = 0, \end{cases} \tag{S5}$$

$$\text{for F}_1\text{:}\quad \begin{cases} (i\omega - \hat{P})E^{\Psi} - t_1 I E^{\eta} - t_2 I G = 0, \\ (i\omega + \hat{P})F^{\Psi\dagger} + t_1 I F^{\eta\dagger} + t_2 I F^{\dagger} = 0, \end{cases} \tag{S6}$$

$$\text{for F}_2\text{:}\quad \begin{cases} (i\omega - \hat{P})E^{\widetilde{\Psi}} - t_3 I G - t_4 I E^{\widetilde{\eta}} = 0, \\ (i\omega + \hat{P})F^{\widetilde{\Psi}\dagger} + t_3 I F^{\dagger} + t_4 I F^{\widetilde{\eta}\dagger} = 0, \end{cases} \tag{S7}$$

$$\text{for S}_1\text{:}\quad \begin{cases} (i\omega - \hat{C}^{(1)})E^{\eta} + i\Delta_1\sigma_y F^{\eta\dagger} - t_1 I E^{\Psi} = 0, \\ (i\omega + \hat{C}^{(1)})F^{\eta\dagger} - i\Delta_1^{*}\sigma_y E^{\eta} + t_1 I F^{\Psi\dagger} = 0, \end{cases} \tag{S8}$$

$$\text{for S}_2\quad \begin{cases} (i\omega - \hat{C}^{(2)})E^{\widetilde{\eta}} + i\Delta_2\sigma_y F^{\widetilde{\eta}\dagger} - t_4 I E^{\widetilde{\Psi}} = 0, \\ (i\omega + \hat{C}^{(2)})F^{\widetilde{\eta}\dagger} - i\Delta_2^{*}\sigma_y E^{\widetilde{\eta}} + t_4 I F^{\widetilde{\Psi}\dagger} = 0. \end{cases} \tag{S9}$$

where I is the 2×2 identity matrix and σ_y is the second Pauli matrix.

The definition of the current through the junction is imposed by the tunnelling Hamiltonian model. In this description, the charge current corresponds to the number of particles travelling from one layer to another, as described by the tunnelling Green's function E. Since the charge current flows across all the layers, it can be obtained from the Green's function at an arbitrary layer. The model presented here focuses on the Green's functions in the central S' layer, such that we express the current in terms of E^{Ψ}. The current consists of the sum of spin-up and spin-down currents, however, since we consider fully polarized ferromagnets, we only need to take the $E^{\Psi}_{\alpha\alpha}$ component into account.

Hence, the Josephson current density (j_y) across the junction is expressed via the Fourier component $E^{\Psi}_{\alpha\alpha}(\mathbf{p};\omega)$ of the off-diagonal Matsubara Green function $E^{\Psi}_{\alpha\beta}(\mathbf{p};\tau_1,\tau_2) = -\langle T_\tau \hat{\psi}_{\mathbf{p},\alpha}(\tau_1)\hat{\phi}^{\dagger}_{\mathbf{p},\beta}(\tau_2)\rangle$:

$$j_y = -2ev_0 t_2 T \,\mathrm{Im} \sum_{\omega=-\infty}^{\infty} \int_{-\infty}^{\infty} E^{\Psi}_{\alpha\alpha}(\mathbf{p};\omega)\, d\xi, \tag{S10}$$

where v_0 and T_τ are the electron density of states at the Fermi level and the time-ordered product for the imaginary time τ, respectively.

By solving the system above, we find the exact expression for E^{ψ}. We obtain $E^{\psi}_{\downarrow\downarrow}(\mathbf{p};\omega) = 0$, which corresponds to the absence of the spin-down state, resulting from fully spin-polarized ferromagnets. By expanding $E^{\psi}_{\uparrow\uparrow}$ up to seventh order over $t_i \ll T,(i = 1, 2, 3, 4)$ (assuming $h_2, h_1 \ll T \approx T_{c1}$ and $|\Delta_0| \ll T$) we find:

$$\mathrm{Im}\sum_{\omega=-\infty}^{\infty}\int_{-\infty}^{\infty} E^{\psi}_{\uparrow\uparrow}(\mathbf{p};\omega)d\xi = 4t_1^2 t_2 t_3^2 t_4^2 \mathrm{Im}[\Delta_1\Delta_2^*](h_1\sin\theta_1)(h_2\sin\theta_2)(a - b|\Delta_0|^2),$$

where the coefficients are given by

$$\begin{aligned} a &= -\sum_{\omega=-\infty}^{\infty}\int_{-\infty}^{\infty}\frac{\omega^2}{(i\omega-\xi)^4(i\omega-\xi)^2[(i\omega+\xi)(i\omega-\xi)-|\Delta_1|^2]^4}d\xi \\ &= \sum_{\omega>0}\frac{\pi\omega^2}{8|\Delta_1|^{14}}\Bigg[-640\omega - \frac{16|\Delta_1|^4}{\omega^3} + \frac{4|\Delta_1|^6}{\omega^5} \\ &\qquad + \frac{640\omega^8 + 2240\omega^6|\Delta_1|^2 + 2816\omega^4|\Delta_1|^4 + 1452\omega^2|\Delta_1|^6 + 231|\Delta_1|^8}{(\omega^2+|\Delta_1|^2)^{7/2}}\Bigg], \end{aligned}$$

$$\begin{aligned} b &= \sum_{\omega=-\infty}^{\infty}\int_{-\infty}^{\infty}\frac{2\omega^2}{(i\omega+\xi)^3(i\omega-\xi)^5[(i\omega+\xi)(i\omega-\xi)-|\Delta_1|^2]^4}d\xi \\ &= \sum_{\omega>0}\frac{\pi\omega^2}{16|\Delta_1|^{16}}\Bigg[4480\omega + \frac{160|\Delta_1|^4}{\omega^3} - \frac{64|\Delta_1|^6}{\omega^5} + \frac{15|\Delta_1|^8}{\omega^7} \\ &\qquad - \frac{4(1128\omega^8 + 3920\omega^6|\Delta_1|^2 + 4940\omega^4|\Delta_1|^4 + 2574\omega^2|\Delta_1|^6 + 429|\Delta_1|^8)}{(\omega^2+|\Delta_1|^2)^{7/2}}\Bigg]. \end{aligned}$$

such that the Josephson current density (S10) becomes

$$j_y = 8ev_0 t_1^2 t_2^2 t_3^2 t_4^2 |\Delta_1|^2 T(h_1\sin\theta_1)(h_2\sin\theta_2)(a - b|\Delta_0|^2)\sin\varphi. \qquad \text{(S11)}$$

Note that the temperature dependence of the critical current in the absence of the singlet superconductivity ($|\Delta_0| = 0$) may be non-monotonous, similar to the results obtained by Eschrig and Löfwander (*11, 12*) for the triplet supercurrents in a half-metallic Josephson junction with spin-active interfaces.

At low temperatures, $T \ll T_{c2}$ and $|\Delta_1| \gg T$, the coefficients a and b reduce to

$$a \approx \frac{7\zeta(3)}{16\pi^2|\Delta_1|^8 T^3}, \qquad b \approx \frac{465\zeta(5)}{512\pi^4|\Delta_1|^8 T^5}.$$

where ζ is the Riemann zeta function.

At a temperature of $T \approx T_{c0}$, the condition above is satisfied when $|\Delta_1(T_{c0})| >> T_{c0}$ (i.e. $T_{c1} >> T_{c0}$). In this case, the Josephson current density is

$$j_y = \frac{56\zeta(3)ev_0 t_1^2 t_2^2 t_3^2 t_4^2 h_1 h_2 \sin\theta_1 \sin\theta_2}{16\pi^2 |\Delta_1|^6 T^2}\left(1 - \frac{465\zeta(5)}{224\pi^2\zeta(3)}\frac{|\Delta_0|^2}{T^2}\right)\sin\varphi. \qquad (S12)$$

The divergence of the critical current in eq. (S12) at low temperature is related to the expansion over t and should be cut off at $T \approx t$. This shows that the singlet superconductivity suppresses the triplet Josephson current. Superconductivity in the central S' layer suppresses the triplet component of the anomalous Green's function $F^\dagger$. The fourth term in the expansion of $F^\dagger$ over t is

$$F_{11}^\dagger(\mathbf{p};\omega) = \frac{\alpha_1 t_1^2 t_2^2 \Delta_1^* + \alpha_2 t_3^2 t_4^2 \Delta_2^*}{(i\omega - \xi)(i\omega + \xi)}\left(1 + \frac{|\Delta_0|^2}{\omega^2 + \xi^2}\right)^{-2}, \qquad (S13)$$

with coefficients $\alpha_{1,2} = d_{1,2}/\{(i\omega - \xi)(i\omega + \xi)\}$, $d_{1,2} = \left[\hat{A}_{1,2}\hat{I}\{i\omega - \hat{C}^{(1,2)}\}^{-1}\right]_{11}$ and $\hat{A}_{1,2} = \left[\{i\omega + \hat{C}^{(1,2)}\} + |\Delta_{1,2}|^2 \hat{I}\{i\omega - \hat{C}^{(1,2)}\}^{-1}\hat{I}\right]^{-1}$.

We conclude that the suppression of the triplet component by the singlet superconducting correlations in the central S' layer results in damping of the Josephson current through a $S_1/F_1/S'/F_2/S_2$ Josephson junction. In the case of a symmetric junctions with $t_1 = t_2 = t_3 = t_4 = t$ and $h_1 = h_2 = h$, for temperature $T_{c1} >> T_{c0}$ with $T \approx T_{c0}$, the Josephson current is

$$j_y = j_0\left(\frac{t}{T_{c0}}\right)^8\left(\frac{h}{T_{c0}}\right)^2\left(\frac{T_{c0}}{|\Delta_1|}\right)^6\left(\frac{T_{c0}}{T}\right)^2\left(1 - \beta\frac{|\Delta_0|^2}{T^2}\right)\sin\theta_1\sin\theta_2\sin\varphi, \qquad (S14)$$

with $j_0 = 56\zeta(3)ev_0T_{c0}^2/(16\pi^2)$ and $\beta = 465\zeta(5)/\{224\pi^2\zeta(3)\} \approx 0.2$.
